# The Health Benefits of Fruits and Vegetables

**DOI:** 10.3390/foods9030369

**Published:** 2020-03-23

**Authors:** Mercedes del Río-Celestino, Rafael Font

**Affiliations:** Agri-Food Laboratory, Council of Agriculture, Fisheries and Rural Development of Andalusia (CAPDER), 14004 Córdoba, Spain; rafaelm.font@juntadeandalucia.es

**Keywords:** fruits, vegetables, biological studies, processing techniques

## Abstract

We edited this Special Issue with the objective of bringing forth new data on the phytochemicals from vegetables and fruits, which are recommended for their health-promoting properties. Epidemiological, toxicological and nutritional studies suggested an association between fruit and vegetable consumption and lower incidence of chronic diseases, such as coronary heart problems, cancer, diabetes, and Alzheimer’s disease. In this Special Issue, the protective roles (antioxidant and others bioactivities), new sustainable approaches to determine the quality, and the processing techniques that can modify the initial nutritional and antioxidant content of fruits, vegetables and additives have been addressed.

Qualitative and quantitative evaluations of the health-beneficial properties of fruits, vegetables and additives have been addressed in this Special Issue, using highly sensitive techniques as well as in vivo and in vitro models. 

Several biological activities have already been reported for kiwifruit (*Actinidia macrosperma*) cultivars, such as antioxidant, anticancer, anti-inflammatory, and antimicrobial activities. This Special Issue contains the first report on a study supporting the potential anti-hypertensive activities of kiwifruits [1]. The results of this study clearly indicate that the flavonoid-rich extract from *A. macrosperma* shows potential as a food or nutraceutical source of anti-hypertensive agents. Further investigations using experimental animal models and human clinical trials are required to explore the anti-hypertensive properties of *A. macrosperma*. 

*Drosophila* is a reliable model to evaluate the toxicity, genotoxicity and other degenerative processes of food or chemical structures [2]. The results obtained in this eukaryote organism are considered translational and highly specific, as more than 80% of genes related to human disease are homologous in *Drosophila* [3]. Additionally, the proapoptotic capacities against cancer processes have been evaluated through the determination of the cytotoxic, clastogenic, and DNA epigenetic modulator activity against in an in vitro human cancer model (HL60 cell line). 

The results from biological activity showed that onion and garlic induced DNA damage in HL60 by necrosis—in concordance with the cytotoxic and DNA-fragmentation results [4]. The chemo-preventive activity of garlic could be associated with its distinctive organosulfur diallyl disulphide compounds (DADS). Supplementary studies are needed to clarify the cell death pathway against garlic and DADS. 

Important information is added to the agrifood industry as the new data provided in this Special Issue [5] suggest that short-aged fermented black garlic (13 days) has higher biological activities than the longer-fermented ones, and even more than raw white garlic. This could have important industrial and economic consequences. Taking both the physicochemical and biological data, black garlic aged for 1 day was shown to have the best nutraceutical properties. These findings are relevant for black-garlic-processing agrifood companies, as the cost and processing time are significantly reduced to 13 days aging. 

Other studies suggest that freeze-dried Czech beers have no severe potential adverse effects [6]. Moreover, all the substances were able to inhibit tumor cell growth and induce DNA damage in the HL-60 cells at different levels (proapoptotic, single/double strand breaks and methylation status). However, further investigations are needed to clarify the effects of beer to other diets, as well as its important role in the prevention of chronic diseases, which mainly are related to the intake of antioxidants. Despite the promising results obtained for the different freeze-dried beers and their materials, their consumption must be moderated due to the known negative effects induced by alcohol. 

New scientific data have been added in this Special Issue in relation to the biological and nutritional effects that food additives (Riboflavin, Tartrazine, Carminic Acid, Erythrosine, Indigotine, and Brilliant Blue) have on time-related degenerative processes. The overall results support the idea that a high chronic intake of food coloring throughout an entire life is not advisable [7], since the in vitro results in HL-60 cells showed that the tested food colorings increased tumor cell growth but did not induce any DNA damage or modifications in the DNA methylation status at their acceptable daily intake concentrations. More research on the biological effects that different concentrations of food colorings could have in model systems is warranted. 

Caramel (caramel color E150d-class IV: CAR) is one of the most worldwide consumed additives and is produced by heating carbohydrates from vegetable sources (glucose, sucrose, invert sugar, etc.) in the presence of caramelization promoters (ammonia or ammonium in class III and IV, respectively). The results reported that CAR was neither toxic nor genotoxic and showed antigenotoxic effects in *Drosophila* [8]. Moreover, caramel showed chemopreventive activity and modified the methylation status of HL-60 cell line. Nevertheless, much more information about the mechanisms of gene therapies related to epigenetic modulation by food is necessary. 

In this Special Issue, we present a systematic, broad-scale metabolomic investigation of 11 species of dried and fresh edible and medicinal mushrooms [9]. The nutritional component analysis of these selected 11 species suggested that mushrooms contained a wide range of proteins, carbohydrates, amino acids, vitamins, and small molecules. The results showing the chemical components of the selected mushrooms provide fundamental data for the development of functional foods from mushrooms. 

Besides the approaches in improving the scientific work to back-up the results, there is a need and clear evolution in the methodologies too in terms of respect to the environment, with more and more conscious labs using greener alternatives to implement sustainable practices from the field to the lab. Standard wet chemistry analytical techniques currently used to determine plant fiber constituents (as those described above) are costly, time-consuming and destructive. Calibration equations based on Near Infrared Reflectance Spectroscopy confirm that this technology could be very useful for the rapid evaluation of acid detergent fiber content in turnip greens and turnip tops (*Brassica rapa* L. subsp. *rapa*) [10]. 

In addition, the germplasm of *Brassica napus* and *Brassica rapa* evaluated in this Special Issue displayed variability in the fatty acid composition of its seed oil [11]. Further research will be needed for some accessions having seeds with reduced or increased values of erucic acid content, in order to select valuable genotypes that could be used for both nutritional and industrial applications. 

The processing techniques at the industrial scale like pasteurization, concentration, and freezing could also modify the initial nutritional and antioxidant content of citrus juices. In this sense, it has been shown that the juice extraction processes employed have influenced the chemical composition and functional properties of bergamot juice (*Citrus bergamia Risso et Poit*., *Rutaceae*). Results from this study suggest that extracting juice under the screw press extractor process increased the amount of phytochemical content and total antioxidant activity, more so than using an in-line extractor and hand-squeezing juicing process [12]. 

Some recent publications have described the beneficial effects of black garlic in the prevention or improvement of cardiovascular diseases, diabetes, obesity, or cancerigenous processes, among others. Black garlic is obtained from raw garlic through a multi-step heating process at a controlled temperature and humidity during a variable period of time. Toledano-Medina et al. [13] have pointed out that an excessive duration in the heating process is detrimental to the final product. The product’s antioxidant capacity diminishes after reaching a prior maximum value when the process is extended, although the polyphenol content goes on increasing. 

Food and nutrition education, food product development, and marketing efforts are called upon to improve adolescent food choices and make less-processed snack food options more appealing and accessible to diverse consumers. Examples of processing levels of snack food items which have the ability to influence adolescent taste preferences are included in this Special Issue [14]. Ultra-processed and processed foods have a large appeal for adolescents, potentially leading to over consumption and unhealthy snacking decisions. Unprocessed and minimally processed food options are not chosen as frequently as processed and ultra-processed foods when all four processing options are made available to an audience of adolescent children. 

The analyses conducted in this Special Issue have showed differences in the expression levels of carotenoid biosynthetic genes and carotenoid content between different *Cucurbita melo* cultivars [15]. These findings will contribute to a foundation for the elucidation of carotenoid biosynthesis in *C. melo*. In addition, further investigations regarding molecular genetics and enzyme activities may help to identify key genes for improving the carotenoid accumulation in melon fruit. 

In summary, as most of the authors have stated, further research is required in relation to each and every one of the presented papers in the “The Health Benefits of Fruits and Vegetables”—this assures an exciting time for the researchers in this field and for the general public interested in the relationship between vegetables and health.
